# Seroprevalence of typhus group and spotted fever group Rickettsia exposures on Reunion island

**DOI:** 10.1186/s13104-019-4416-8

**Published:** 2019-07-09

**Authors:** Patrick Gérardin, Naël Zemali, Marie Bactora, Guillaume Camuset, Elsa Balleydier, Hervé Pascalis, Vanina Guernier, Corinne Mussard, Antoine Bertolotti, Yatrika Koumar, Florence Naze, Sandrine Picot, Laurent Filleul, Frédéric Pages, Pablo Tortosa, Julien Jaubert

**Affiliations:** 1 INSERM Centre d’Investigation Clinique 1410 Epidémiologie Clinique, Centre Hospitalier Universitaire (CHU), Groupe Hospitalier Sud Réunion, BP 350, 97448 Saint Pierre Cedex, Reunion France; 2 UM 134 PIMIT Processus Infectieux en Milieu Insulaire Tropical, INSERM 1187, CNRS 9192, IRD 249, CYROI, Université de La Réunion, Sainte Clotilde, Reunion France; 3 Laboratoire de Bactériologie, Virologie et Parasitologie, CHU de la Réunion, Saint Pierre, Reunion France; 4 Service des Maladies Infectieuses, CHU de la Réunion, Saint Pierre, Reunion France; 5 Cellule d’Intervention Régionale et d’Epidémiologie, Océan Indien, Santé Publique France, French National Public Health Agency, Saint Denis, Reunion France; 6 Geelong Centre for Emerging Infectious Diseases, Deakin University, Geelong, VIC Australia

**Keywords:** Serology, Immunofluorescence, Seroepidemiologic study, Serosurvey, Stored frozen serum, Murine typhus, *Spotted fever*, Zoonosis, Prevalence, General population

## Abstract

**Objective:**

Murine typhus has been increasingly reported on Reunion island, Indian ocean, following documentation of eight autochthonous infections in 2012–2013. We conducted a serosurvey to assess the magnitude of the seroprevalence of rickettsioses in the population. Two hundred and forty-one stored frozen sera taken from the 2009 Copanflu-RUN cohort were analysed using an immunofluorescence assay allowing to distinguish typhus group (TGR) and spotted fever group *Rickesttsiae* (SFGR). Seropositivity was defined for a dilution titre of Rickettsia IgG antibodies ≥ 1:64. Seroprevalence was weighted to account for the discrepancy between the Copanflu-RUN subset and the general population, as to infer prevalence at community level. Prevalence proportion ratios (PPR) were measured using log-binomial models.

**Results:**

The weighted seroprevalences of typhus group rickettsioses and spotted fever group rickettsioses were of 12.71% (95% CI 8.84–16.58%) and 17.68% (95% CI 13.25–22.11%), respectively. Pooled together, data suggested that a fifth of the population had been exposed at least to one *Rickettsia* group. Youths (< 20 years) were less likely seropositive than adults (adjusted PPR 0.13, 95% CI 0.01–0.91). People living in the western dryer part of the island were more exposed (adjusted PPR 2.53, 95% CI 1.07–5.97). Rickettsioses are endemic on Reunion island and circulated before their first identification as murine typhus in year 2011. Surprisingly, since isolation *of Rickettsia africae* from *Amblyomma variegatum* in year 2004 or isolation of *Rickettsia felis* from *Amblyomma loculosum*, no autochthonous cases of African tick-bite fever or flea-borne spotted fever has yet been diagnosed.

**Electronic supplementary material:**

The online version of this article (10.1186/s13104-019-4416-8) contains supplementary material, which is available to authorized users.

## Introduction

*Rickettsiae* are Gram negative obligate intracellular bacteria that include several zoonotic pathogens distributed worldwide [1]. The *Rickettsia* genus is divided phylogenetically into four distinct groups [2].

*Typhus group Rickettsiae* (TGR) consist of *R. prowazekii* and *R. typhi*, the agents responsible for epidemic and murine typhus, respectively. *Rickettsia typhi* is usually maintained in rodents and transmitted by the rat flea *Xenopsylla cheopis* [1]. Invasive rodents, specifically the black rats (*Rattus rattus*), and the brown rats (*Rattus norvegicus*), serve as primary reservoirs. Humans are infected through contamination of disrupted skin, respiratory tract, or conjunctivae with infected flea faeces. Symptoms are non-specific and cover a large spectrum from mild to severe illness. Even though TGR pathogens are ubiquitous, their burden is higher in tropical regions [3].

*Spotted Fever Group Rickettsiae* (SFGR) encompass 20 *Rickettsia* species (*R. rickettsii*, *R. conorii*, *R. africae*, etc.…) mostly transmitted by ticks (*Dermacentor* spp., *Amblyomma cajennense* and *Rhipicephalus sanguineus*, etc.…) [2]. SFGR multiply in endothelia of almost all organs causing vasculitis. SFGR infections may result into mild to severe and potentially fatal disease.

The ancestral group consists of *R. canadensis* and *R. bellii*, and the transitional group is composed of *R. akari, R. australis* and now *R. felis* (formerly classified in SFGR). Among these, only the transitional group is pathogenic to humans [2].

After a reporting of eight autochthonous cases of TGR infection on Reunion island between January 2011 and January 2013 and a subsequent case series [4, 5], we conducted a serosurvey using stored frozen human sera dated 2009 to assess the magnitude of exposure to *Rickettsiae* in the community.

## Main text

### Methods

#### Setting and population

La Réunion is a small tropical island (2512 km^2^), located in the South Western Indian ocean, 700 km east of Madagascar. Its landscape gives rise to contrasted climates with a mountainous centre separating a humid windward east coast from a dry leeward west coast. The lower portion of this latter consists of a savannah where introduced rodents (*R. rattus* and *R. norvegicus*) display distinctly higher flea infestation than in the humid portions of the island [6]. Most of the 816,000 inhabitants present in 2009 lived in the coastal area where the cities lie. The structure by age, gender, and microregion (i.e., 4 administrative regional subdivisions) of the general population is presented in Additional file 1: Table S1.

The study population was a subset of the CoPanFlu-RUN cohort, dedicated to 2009 pandemic flu [7]. Data pertaining to age, gender, and place of residence were retrieved for each individual and compared to the general population (Additional file 1: Table S1).

#### Serology

Two hundred and forty-one sera were tested using an indirect fluorescent antibody (IFA) assay with commercially available *R. typhi* and *R. rickettsii* antigens (IF0100G, Rickettsia IFA IgG^®^, Focus Diagnostics Inc., Cypress, CA, USA) used as TGR and SFGR antigens [8]. A positive specimen with an IgG titre ≥ 1:64 was considered as indicative of a previous infection. Each positive sample was further diluted to 1:128 for a more stringent definition. The sera were checked for *Coxiella burnetii* [9], another Gram negative obligate bacterium, formerly classified within the Rickettsiaceae family and now assigned to the *Coxiellaceae*.

#### Statistical analysis

Statistical analysis was performed using Stata 14.2^®^ (StataCorp, College Station; Texas, USA). The seroprevalence rate was measured as crude (raw or unadjusted) and weighted to account for the disproportions between the CoPanFlu-RUN subset and the general population. Associations between positive specimens for TGR or SGR and age, gender and geographic subdivision were determined using weighted Chi square tests. Survey-adjusted log-binomial regression models were used to estimate prevalence proportion ratios (PPR) and 95% confidence intervals (95% CI). A *P* value < 0.05 was considered significant.

### Results

The raw seroprevalence of TGR infections at a cut-off of 1:64 was of 16.18% (39/241) and the weighted seroprevalence of 12.71% (95% CI 8.84–16.58%). These figures were respectively of 9.13% (22/241) and 8.24% (95% CI 5.04–11.43%) with the more stringent dilution of 1:128.

In comparison, the raw seroprevalence of SFGR infections at a cut-off of 1:64 was of 18.67% (45/241) and the weighted seroprevalence of 17.68% (95% CI 13.25–22.11%). These figures were respectively of 12.86% (31/241) and 10.44% (95% CI 6.89–13.99%) at a dilution of 1:128.

Altogether, these data revealed that a fifth of the population of Reunion island exhibited a positive serology [22.41% (54/241); 18.15%; 95% CI 13.67–22.63%], indicative of a previous infection or exposure with either one of the two *Rickettsi*ae groups.

Both TGR and SFGR infections were less frequent among youths (< 20 years) than among adults, although prevalence did not progress with age through adulthood (Table 1 and Additional file 1: Table S2, Table 2 and Additional file 1: Table S3). Women and men were similarly exposed. The population living in the western dryer microregion was respectively twofold and threefold more likely to have been exposed to TGR and SFGR than people living in the South.

**Table 1:** Factors independently associated with typhus group Rickettsiae (TGR) seropositivity in multivariate analysis, Reunion island, 2009 (n = 241)

*Rickettsia typhi IgG *≥* 1:64*				
Variables	n	%	Adjusted PPR	95% CI
Age				
< 20 years	1/24	2.07	0.13*	0.01–0.91
20–39 years	14/77	16.35	1	–
40–59 years	19/102	13.44	0.81	0.40–1.64
≥ 60 years	5/38	10.28	0.51	0.18–1.42
Gender				
Female	19/136	11.69	0.74	0.40–1.35
Male	20/105	17.19	1	–
Microregion**				
North	0/17	0.00	NA	–
South	8/80	6.76	1	–
West	20/78	23.22	2.53*	1.07–5.97
East	11/66	17.74	2.21	0.85–5.70

Data are numbers (n), weighted seropositive rates (%), adjusted prevalence proportion ratios (PPR), and 95% confidence intervals (95% CI)

*NA* not assessed

*P* values linked to variable names are given for overall design-based Pearson chi2 tests

*P* values linked to PPR are given for within-each-category Wald tests

* *P* < 0.05

** *P* < 0.01

**Table 2:** Factors independently associated with spotted fever group Rickettsiae (SFGR) seropositivity in multivariate analysis, Reunion island, 2009 (n = 241)

*Rickettsia rickettsii IgG *≥* 1:64*				
Variables	n	%	Adjusted PPR	95% CI
Age				
< 20 years	0/24	0.00	NA**	
20–39 years	13/77	18.84	1	–
40–59 years	23/102	21.88	1.22	0.55–2.69
≥ 60 years	9/38	21.91	1.20	0.46–3.15
Gender				
Female	25/136	17.92	1.05	0.61–1.82
Male	20/105	16.61	1	
Microregion				
North	2/17	14.63	1.62	0.30–8.72
South	6/80	5.73	1	–
West	26/78	30.07	3.61*	1.32–9.87
East	11/66	17.01	2.10	0.68–6.42

Data are numbers (n), weighted seropositive rates (%), adjusted prevalence proportion ratios (PPR), and 95% confidence intervals (95% CI)

*NA* not assessed

*P* values linked to variable names are given for overall design-based Pearson chi2 tests

*P* values linked to PPR are given for within-each-category Wald tests

* *P* < 0.05

** *P* < 0.01

At the cut-off value of 1:64, 30 out of the 54 positive sera (55.5%) were positive both to TGR and SFGR. Seven positive sera (13.0%) also reacted with *C. burnetii* (2 with both TGR and SFGR, 3 with SFGR alone, 2 with TGR alone).

### Discussion

*Rickettsiae* are widely distributed in tropical areas and have been previously reported in seabird ticks, rodent ticks and rodent fleas including on Reunion island [10–12].

Herein, we provide the first serological evidence of a high magnitude exposure of the population living on Reunion island to pathogenic *Rickettsiae*.

Given our serosurvey was conducted on sera sampled nearly 2 years before the clinical identification of the first autochthonous cases and that the youngest subjects were relatively spared, we suggest that the TGR and SFGR antibodies are long-lived and rickettsioses have been endemic on Reunion island, as previously hypothesized for the Amazon Basin of Peru [13]. Together, these elements support the idea that *Rickettsia* infections are unrecognized or misdiagnosed and account for a substantial proportion of fevers of unknown origin, as suggested by a case report of prior murine typhus infection in a traveller returning from Reunion island [14].

Another point to highlight is that seropositive subjects live predominantly on the western dryer part of the island, which is both coherent with the spatial distributions of rat fleas and clinical cases [4, 5]. Furthermore, *Rickettsiae* from different groups (*R. typhi* and *R. felis*) have been identified in rat fleas in this part of the island [12]. Importantly, *R. africae* has been isolated from *Amblyomma loculosum*, a seabird tick in remote areas from human dwellings [11], and its main vector *Amblyomma variegatum*, a cattle tick, has drastically decreased in the western savannah over the last 20 years [15], which makes unlikely the spill over of *R. africae* from both seabirds or cattle to humans. So, we suggest that *R. felis* may be partially responsible of SFGR seropositivity on Reunion island. Interestingly, *R. felis*, a pathogen that share common features of both SFGR and TGR, has recently been identified in *Anopheles gambiae* mosquitoes and other non-hematophagous arthropods, which makes the understanding of its transmission pathways very complex with several potential vectors [16]. Another possibility is that a slight proportion of SFGR positive serologies may have been acquired while travelling abroad given the western population is also putatively the most mobile on the island. Consistent with this latter hypothesis, SFGR are endemic in the south western Indian ocean region, where both African tick-bite fever, spotted fever and flea-borne spotted fever regularly diagnosed [17, 18].

Finally, our findings warrant a larger scale seroepidemiologic study aimed at better assessing the prevalence of exposure to *Rickettsiae* and understanding the transmission pathways of these environmental microbial pathogens. Of note, flea indices reported in rodents from the western portion of the island are heterogeneous geographically, and fleas were found only in rats from the dry savannah at a low altitude. Hence, a better powered size study should better discriminate into the habitats and provide a finer resolution of the current epidemiological pattern.

## Limitations

This study has potential limitations. First, our findings may be prone to resampling bias given the serosurvey is based on stored frozen sera issued from participants sampled for another purpose. Second, serology cross reactions within the *Rickettsiaceae* family and with *Coxiella burnetii* may have precluded the precise identification of the involved pathogens [9]. These things being said, cross reactions with *C. burnetii* were infrequent (13.0%), so we believe that this limitation is unlikely to change the overall magnitude of Reunion island population exposure to *Rickettsiae*. Third, it is worth noting that autochthonous clinical cases of spotted fever have never been reported on Reunion island, and within the *Rickettsia* genus, misclassification may have affected the seroprevalence estimates, given both the likelihood of intra-genus cross reactions and the impossibility to link SFGR antibodies to *R. felis* exposure in our retrospective study.

Notwithstanding, it would be informative to carry out a more specific serosurvey (such as sero-neutralization) in order to identify the pathogens at the species level.

## Additional file

**Additional file 1: Table S1.** Socio-demographic characteristics of the study population and Réunion island population, 2009. *Data are numbers and percentages related to age, gender and residence at both the study population and the community*. **Table S2.** Factors associated with typhus group Rickettssiae (TGR) seropositivity in bivariate analysis, Reunion island, 2009. **Table S3.** Factors associated with Spotted Fever Group Rickettsiae (SFGR) seropositivity in bivariate analysis, Reunion island, 2009. *For Tables S2 and S3, data are numbers, weighted seropositive rates, crude prevalence proportion ratios, and 95% confidence intervals.*

## Data Availability

The dataset is available from the corresponding author on reasonable request.

## Abbreviations

CPP: Comité de Protection des Personnes
IFA: indirect fluorescent antibody (alternatively taken as immunofluorescent assay)
IgG: immunoglobulin G
95% CI: 95% confidence interval
PPR: prevalence proportion ratio
SFGR: spotted fever Group *Rickettsiae*
TGR: typhus group *Rickettsiae*
