# Supplementary material for: Seroprevalence of typhus group and spotted fever group Rickettsia exposures on Reunion island
Source: BMC Res Notes. 2019 Jul 9;12:387. doi: 10.1186/s13104-019-4416-8 (PMC6617902; doi:10.1186/s13104-019-4416-8)
Supplement: Supplementary file 1 — Additional file 1: Table S1. Socio-demographic characteristics of the study population and Réunion island population, 2009. Data are numbers and percentages related to age, gender and residence at both the study population and the community. Table S2. Factors associated with typhus group Rickettssiae (TGR) seropositivity in bivariate analysis, Reunion island, 2009. Table S3. Factors associated with Spotted Fever Group Rickettsiae (SFGR) seropositivity in bivariate analysis, Reunion island, 2009. For Tables S2 and S3, data are numbers, weighted seropositive rates, crude prevalence proportion ratios, and 95% confidence intervals. [file 13104_2019_4416_MOESM1_ESM.docx]

| **Table S1. Socio-demographic characteristics of the study population and Réunion island population, 2009 (n=241)** | | | | | |
| --- | --- | --- | --- | --- | --- |
|  | **Study population** | | **Reunion island population ^†^** | |  |
| **Variables** | **n** | **%** | **N** | **%** | ***P-value*** |
| **Age** |  |  |  |  | < 0.001 |
| <20 years | 24 | 10.0 | 279,585 | 34.2 |  |
| 20-39 years | 77 | 32.0 | 227,738 | 27.9 |  |
| 40-59 years | 102 | 42.3 | 213,165 | 26.1 |  |
| ≥ 60 years | 38 | 15.8 | 95,876 | 11.7 |  |
| **Gender** |  |  |  |  | 0.128 |
| Female | 136 | 56.4 | 420,676 | 51.5 |  |
| Male | 105 | 43.6 | 395,688 | 48.5 |  |
| **Microregion** |  |  |  |  | < 0.001 |
| North | 17 | 7.1 | 198,013 | 24.3 |  |
| South | 80 | 33.2 | 289,896 | 35.5 |  |
| West | 78 | 32.4 | 209,835 | 25.7 |  |
| East | 66 | 27.4 | 118,620 | 14.5 |  |
| Data are numbers (n and N) and percentages. **^†^** 2009 Insee census (Institut national de Statistiques et d'études économiques) census based on municipal data (population of 816,364 inhabitants). NA: not assessed. | | | | | |

| **Table S2. Factors associated with Typhus Group Rickettssiae (TGR) seropositivity in bivariate analysis, Reunion island, 2009 (n=241)** | | | | |  |
| --- | --- | --- | --- | --- | --- |
| ***Rickettsia typhi IgG ≥ 1:64*** | | | | |  |
| **Variables** | **N** | **%** | **Crude PPR** | **95% CI** |  |
| **Age** |  |  |  |  |  |
| <20 years | 1/24 | 2.07 | 0.23 | 0.03 - 1.66 |  |
| 20-39 years | 14/77 | 16.35 | 1 | - |  |
| 40-59 years | 19/102 | 13.44 | 1.02 | 0.54 - 1.91 |  |
| ≥ 60 years | 5/38 | 10.28 | 0.72 | 0.28 - 1.86 |  |
| **Gender** |  |  |  |  |  |
| Female | 19/136 | 11.69 | 1.08 | 0.60 - 1.93 |  |
| Male | 20/105 | 17.19 | 1 |  |  |
| **Microregion ***** |  |  |  |  |  |
| North | 0/17 | 0.00 | NA |  |  |
| South | 8/80 | 6.76 | 1 | - |  |
| West | 20/78 | 23.22 | 3.43 ****** | 1.40 - 8.42 |  |
| East | 11/66 | 17.74 | 2.62 | 0.99 - 6.88 | |
| Data are numbers (n), weighted seropositive rates (%), crude prevalence proportion ratios (PPR), and 95% confidence intervals (95% CI). NA: not assessed. *P* values linked to variable names are given for overall design-based Pearson chi2 tests. *P* values linked to PPR are given for within-each-category Wald tests. * *P* <0.05. ** *P* <0.01. | | | | | |

| **Table S3. Factors associated with Spotted Fever Group Rickettsiae (SFGR) seropositivity in bivariate analysis, Reunion island, 2009 (n=241)** | | | | | |
| --- | --- | --- | --- | --- | --- |
| ***Rickettsia rickettsii IgG ≥ 1:64*** | | | | | |
| **Variables** | **n** | | **%** | **Crude PPR** | **95% CI** |
| **Age** | |  |  |  |  |
| <20 years | | 0/24 | 0.00 | NA ** |  |
| 20-39 years | | 13/77 | 18.84 | 1 | - |
| 40-59 years | | 23/102 | 21.88 | 1.33 | 0.72 - 2.47 |
| ≥ 60 years | | 9/38 | 21.91 | 1.40 | 0.65 - 2.99 |
| **Gender** | |  |  |  |  |
| Female | | 25/136 | 17.92 | 0.96 | 0.56 - 1.64 |
| Male | | 20/105 | 16.61 | 1 |  |
| **Microregion** | |  |  |  |  |
| North | | 2/17 | 14.63 | 1.57 | 0.39 - 7.14 |
| South | | 6/80 | 5.73 | 1 | - |
| West | | 26/78 | 30.07 | 4.44 ** | 1.93 - 10.22 |
| East | | 11/66 | 17.01 | 2.22 | 0.86 - 5.70 |
| Data are numbers (n), weighted seropositive rates (%), crude prevalence proportion ratios (PPR), and 95% confidence intervals (95% CI). NA: not assessed. *P* values linked to variable names are given for overall design-based Pearson chi2 tests. *P* values linked to PPR are given for within-each-category Wald tests. * *P* <0.05. ** *P* <0.01. | | | | | |
